# Berberine induces oxidative DNA damage and impairs homologous recombination repair in ovarian cancer cells to confer increased sensitivity to PARP inhibition

**DOI:** 10.1038/cddis.2017.471

**Published:** 2017-10-05

**Authors:** Dong Hou, Guangwei Xu, Caibo Zhang, Boxuan Li, Junchao Qin, Xiaohe Hao, Qiao Liu, Xiyu Zhang, Jinsong Liu, Jianjun Wei, Yaoqin Gong, Zhaojian Liu, Changshun Shao

**Affiliations:** 1 Department of Molecular Medicine and Genetics, Ministry of Education Key Laboratory of Experimental Teratology, Shandong University School of Medicine, Jinan, China; 2 Department of Pathology, The University of Texas MD Anderson Cancer Center, Houston, TX, USA; 3 Department of Pathology, Northwestern University School of Medicine, Chicago, IL, USA; 4 Department of Cell Biology, Shandong University School of Medicine, Jinan, China; 5 Jiangsu Key Laboratory of Stem Cells and Biomaterials, Institutes for Translational Medicine, Soochow University, 199 Ren Ai Road, Suzhou, Jiangsu 215123, China

## Abstract

Many cancer drugs exert their therapeutic effect by inducing oxidative stress in the cancer cells. Oxidative stress compromises cell survival by inflicting lesions in macromolecules like DNA. Cancer cells rely on enhanced antioxidant metabolism and increased DNA repair function to survive oxidative assault. PARP1, a protein that senses DNA-strand breaks and orchestrates their repair, has an important role in the repair of oxidative DNA damage. Berberine, an alkaloid compound present in many herbal plants, is capable of inducing oxidative DNA damage and downregulating homologous recombination repair (HRR) in cancer cells. In this study, we demonstrated that berberine and PARP inhibitor niraparib have a synthetic lethal effect on ovarian cancer cells. Oxidative DNA damage was greatly induced by berberine in ovarian cancer cells. In addition, the level of RAD51 and the capacity of HRR were also reduced by berberine. Correspondingly, PARP became hyperactivated in response to berberine treatment. Cancer cells treated with berberine and niraparib in combination exhibited greatly increased apoptosis and remarkably reduced tumor growth *in vivo*. Together, the results indicate that by inducing oxidative DNA damage and downregulating HRR in cancer cells berberine is able to further sensitize cancer cells to PARP inhibition. Our findings demonstrate a potential therapeutic value of combined application of berberine and PARP inhibitors in ovarian cancer treatment.

Ovarian cancer is one of the most common causes of death among women with gynecologic malignancies. About 60−70% patients with ovarian cancer were diagnosed in their advanced stage and overall 5-year survival is ~30%.^1^ Debulking surgery followed by platinum–taxane-based chemotherapy is the standard of care for patients with advanced-stage ovarian cancer. However, despite an encouraging response rate of 65–80% to first-line chemotherapy, most patients relapse with chemoresistant disease.^2^ Late diagnoses and relapse with chemoresistant disease lead to a lack of remarkable improvements in the cure rate over the past 30 years.^3^

Berberine, an isoquinoline alkaloid component in several medicinal herbs, has been shown to have antimicrobial, anti-inflammatory, anti-diabetic, anti-angiogenesic and cholesterol-lowering effects.^4, 5^ In addition, berberine possesses antitumor activities against various tumor cells.^6, 7, 8, 9^ Berberine has also been shown to suppress tumor growth in xenograft models.^9, 10, 11, 12, 13^ Among others, induction of oxidative stress and DNA damage have been reported to mediate apoptosis and cell cycle arrest.^6, 8^ In addition to acting as a genotoxicant, berberine can also radiosensitize cancer cells by downregulating RAD51, a key factor in homologous recombination repair (HRR).^14^

By sensing DNA-strand breaks and orchestrating base excision repair, PARP1 has an important role in the repair of oxidative DNA damage.^15, 16, 17^ PARP1 also becomes hyperactivated in cells with defective HRR,^18, 19^ and HRR-defective cells are particularly sensitive to PARP inhibition.^18, 19, 20^ Because berberine can induce oxidative stress and downregulate HRR, conditions in which cells are more reliant on PARP1 for survival, we speculated that berberine-treated ovarian cancer cells might be more sensitive to PARP inhibition. In this study, we tested the effect of combined application of berberine and PARP inhibitor on ovarian cancer cells. We observed increased levels of oxidative stress and DNA damage in ovarian cancer cells treated with berberine. Moreover, RAD51 as well as HR were also downregulated by berberine. As expected, apoptosis was greatly induced and tumor growth was remarkably reduced when berberine and PARP inhibitor were applied in combination. Our findings may bear implications in designing cancer therapy strategies involving two or more drugs.

## Results

### Berberine induces oxidative stress and DNA damage in ovarian cancer cells

**Figure 1:**
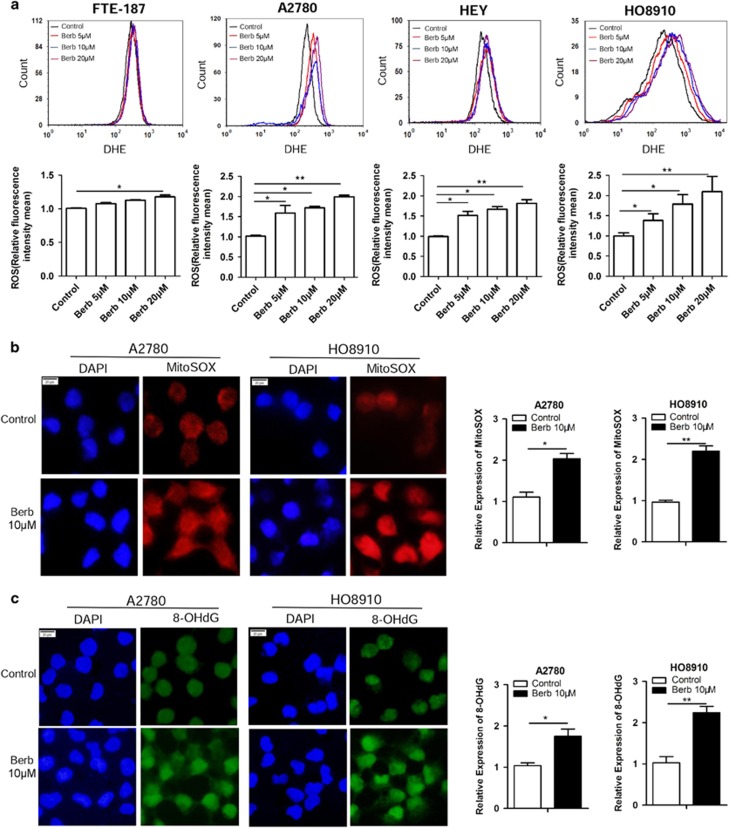
Berberine increases oxidative stress in ovarian cancer cells. (**a**) A2780, HEY, HO8910 and FTE-187 cells were treated with different concentrations of berberine for 48 h. ROS generation was measured using oxidation-sensitive fluorescent probe (DHE) by flow cytometry. Means and S.D.s of three repeats were shown at the bottom. (**b**) Left: immunofluorescence staining of MitoSOX in A2780 and HO8910 treated with berberine (10 *μ*M) for 48 h. Right: quantification of MitoSOX expression in A2780 and HO8910 treated with berberine (10 *μ*M) for 48 h. (**c**) Left immunofluorescence staining of 8-OHdG in A2780 and HO8910 treated with berberine (10 *μ*M) for 48 h. Right: quantification of 8-OHdG expression in A2780 and HO8910 treated with berberine (10 *μ*M) for 48 h. Immunofluorescence intensities were quantified by ImageJ. *P<0.05, **P<0.01

Berberine has been reported to promote the generation of reactive oxygen species (ROS)^21, 22^ and induce DNA damage in human cancer cells.^6^ We, therefore, measured the level of ROS in ovarian cancer cells treated with different concentrations of berberine for 48 h. We used the superoxide-sensitive dihydroethidium (DHE) as a probe and analyzed the ROS level using flow cytometry. As shown in Figure 1a, the ROS level was significantly increased in berberine-treated A2780, HEY and HO8910 cells at all three concentrations tested. In contrast, the ROS level was slightly increased only at the highest concentration in immortalized fallopian tube epithelial cells (FTE-187). MitoSOX, an indicator of mitochondrial superoxide, also yielded more intense staining in berberine-treated A2780 and HO8910 cells under a fluorescence microscope (Figure 1b). Consistently, the level of 8-OHdG, a common oxidized base damage, was higher in berberine-treated cells when compared with control (Figure 1c).

**Figure 2:**
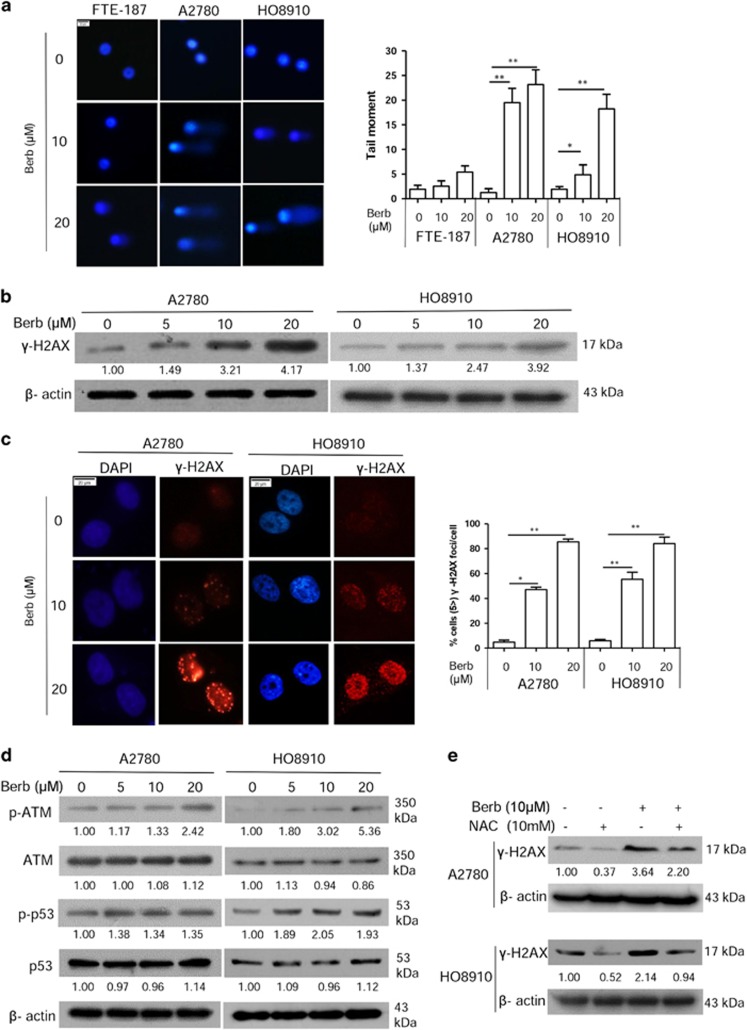
Berberine increases DNA damage in ovarian cancer cells. (**a**) FTE-187, A2780 and HO8910 cells were treated with berberine for 48 h and run in alkaline comet assay; tail moment is calculated as percent DNA in the tail multiplied by the tail length. (**b**) Western blotting analysis of *γ*-H2AX protein levels in A2780 and HO8910 treated with berberine for 48 h. (**c**) Immunofluorescence staining of *γ*-H2AX in A2780 and HO8910 treated with berberine for 48 h. Left: representative examples of immunofluorescence staining of *γ*-H2AX foci. Scale bar, 20 *μ*m. Right: distribution of cells with at least five *γ*-H2AX foci. For each group 500 cells were counted. Shown are the averages and SD of three repeats. (**d**) Western blotting analysis of p-ATM, ATM, p-p53 and p53 protein levels in A2780 and HO8910 cells treated with berberine for 48 h. (**e**) Western blotting analysis of *γ*-H2AX protein levels in A2780 and HO8910 cells treated with berberine (10 *μ*M) or NAC (10 mM), alone or in combination for 48 h. The numbers shown below bands were folds of band intensities relative to control. Band intensities were quantified by ImageJ and normalized to *β*-actin. Data are expressed as a fold change relative to the control. *P<0.05, **P<0.01

We next measured the level of DNA damage in berberine-treated ovarian cancer cells using an alkaline comet assay. As shown in Figure 2a, berberine treatment at 10 or 20 *μ*M for 48 h led to a great increase in the generation of strand breaks in both cell lines tested. In contrast, much less DNA damage was induced in FTE-187 cells. In addition, the phosphorylation of H2AX at ser139, or *γ*-H2AX, a marker of DSBs, was also elevated in A2780 and HO8910 cells treated with berberine for 48 h, as indicated by western blot and immunofluorescence analyses (Figures 2b and c). As expected, ATM and p53 were both activated in response to berberine treatment (Figure 2d), suggesting that DNA damage response was initiated in berberine-treated cells. Importantly, the *γ*-H2AX level can be attenuated by antioxidant N-acetylcysteine (NAC) in berberine-treated cells (Figure 2e), indicating that the increase in ROS is partially responsible for the elevation of DSBs.

### Berberine downregulates RAD51 and homologous recombination in ovarian cancer cells

**Figure 3:**
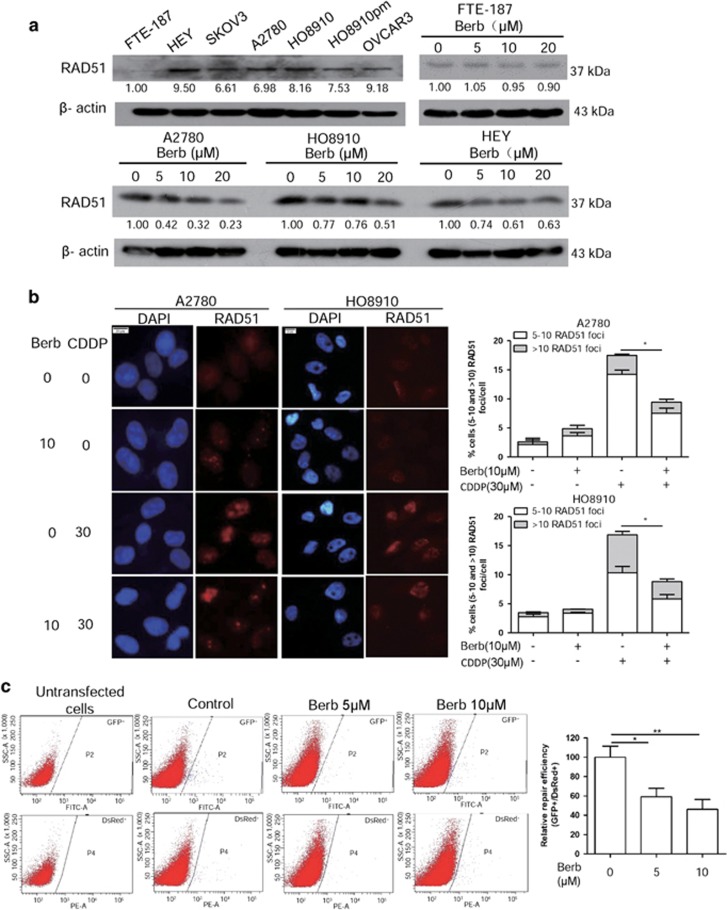
Berberine downregulates RAD51 and homologous recombination in ovarian cancer cells. (**a**) Western blotting analysis of RAD51 protein levels in ovarian cancer cells and in A2780, HEY, HO8910 and FTE-187 cells treated with different concentrations of berberine for 48 h. The numbers shown below bands were folds of band intensities relative to control. Band intensities were quantified by ImageJ and normalized to *β*-actin. Data are expressed as a fold change relative to the control. (**b**) Immunofluorescence staining of RAD51 in A2780 and HO8910 treated with berberine (10 *μ*M) alone or in combination with CDDP (30 *μ*M) for 48 h. Left: representative examples of immunofluorescence staining of RAD51 foci. Scale bar, 20 *μ*m. Right: distribution of cells with at least five RAD51 foci. For each group 500 cells were counted. Shown are the averages and S.D. of three repeats. (**c**) Inhibition of HR repair by berberine. A2780 cells were treated with different concentrations of berberine for 48 h and were then co-transfected with 2 *μ*g linearized GFP HR reporter plasmids and 0.1 *μ*g of the DsRed expression vector for 72 h. The DsRed was used to normalize for the differences in transfection efficiency. Cells were analyzed on a green (P2) *versus* red (P4) fluorescence plot. The numbers of GFP^+^ and DsRed^+^ cells were determined by flow cytometry. The ratio of GFP^+^ to DsRed^+^ cells was used as a measure of repair efficiency. Left: typical FACS traces, P2 and P4 represent green and red fluorescence plot, respectively. Right: quantitative summary of HR efficiency in A2780 cells after treatment with berberine. *P<0.05, **P<0.01

RAD51 is a key factor in HRR. Upregulation of RAD51 in cancer cells was shown to be associated with increased chemoresistance.^23^ We previously reported that berberine could radiosensitize human esophageal cancer cells by downregulating RAD51.^14^ We therefore also measured RAD51 protein level in ovarian cancer cells treated with berberine. As shown in Figure 3a, the RAD51 levels are higher in ovarian cancer cells than in FTE-187 cells, and berberine treatment led to a significant downregulation of RAD51 in A2780, HEY and HO8910 cells. Interestingly, berberine had no effect on RAD51 expression in FTE-187 cells.

Formation of RAD51 foci reflects a key step in HRR. As shown in Figure 3b, the number of RAD51 foci induced by cisplatin (CDDP) in A2780 and HO8910 cells was significantly reduced when cancer cells were co-treated with berberine. We next determined whether the downregulation of RAD51 by berberine would lead to changes in the capacity of HRR using a GFP-based reporter assay. As shown in Figure 3c, DNA repair efficiency was decreased significantly in berberine-treated A2780 cells compared with untreated cells. These results suggest that berberine can significantly impair HRR in ovarian cancer cells.

### Berberine activates PARP in ovarian cancer cells

**Figure 4:**
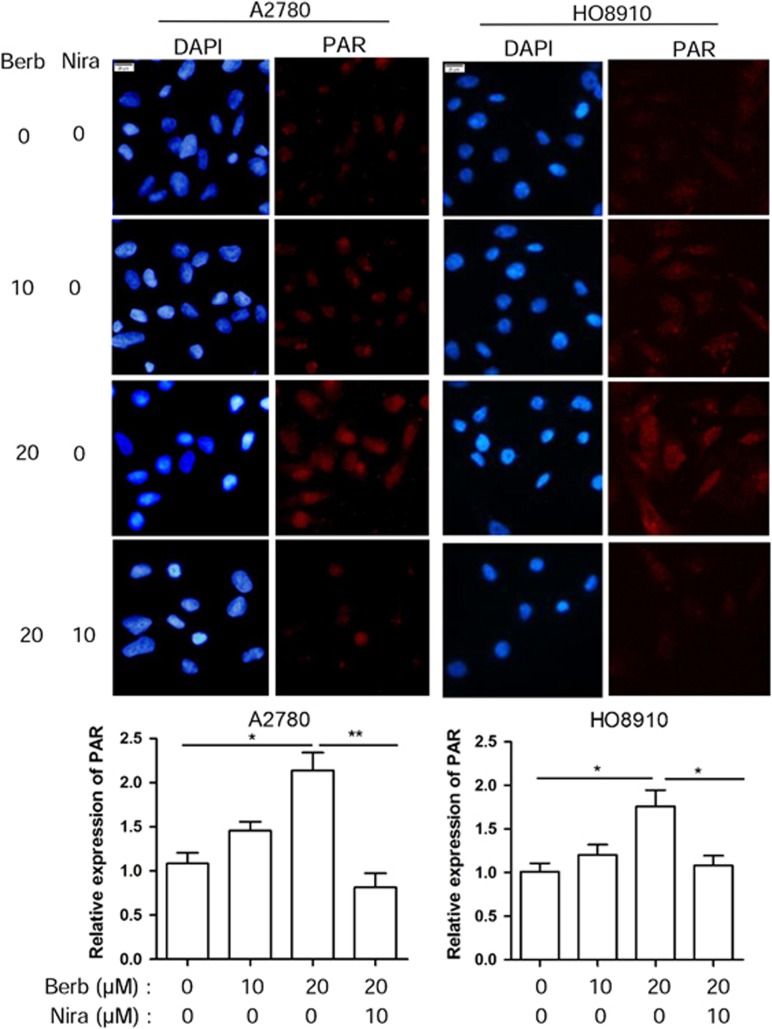
Berberine activates PARP1 in ovarian cancer cells. PAR synthesis is detected by immunofluorescence staining in A2780 and HO8910 treated with different concentration of berberine alone or in combination with niraparib (10 *μ*M) for 48 h. Top: representative examples of immunofluorescence staining of PAR. Scale bar, 20 *μ*m. Bottom: quantification of PAR level in A2780 and HO8910. Immunofluorescence intensities were quantified by ImageJ. *P<0.05, **P<0.01

Oxidative stress is a well-known inducer of PARP1 activation.^16, 17^ Downregulation of HR is also associated with increased activation of PARP1.^24^ Because berberine treatment leads to both an increased oxidative stress and a downregulation of HR, we expected that PARP1 would become hyperactivated after treatment with berberine. We performed immunofluorescence of poly(ADP-ribose) or PAR, a product of PARP activation, in berberine-treated cancer cells. As shown in Figure 4, the amount of PAR was greatly increased when A2780 or HO8910 cells were treated with berberine for 48 h. This increase could be blocked by PARP inhibitor niraparib.

### Berberine and niraparib act synergistically to induce apoptotic cell death

**Figure 5:**
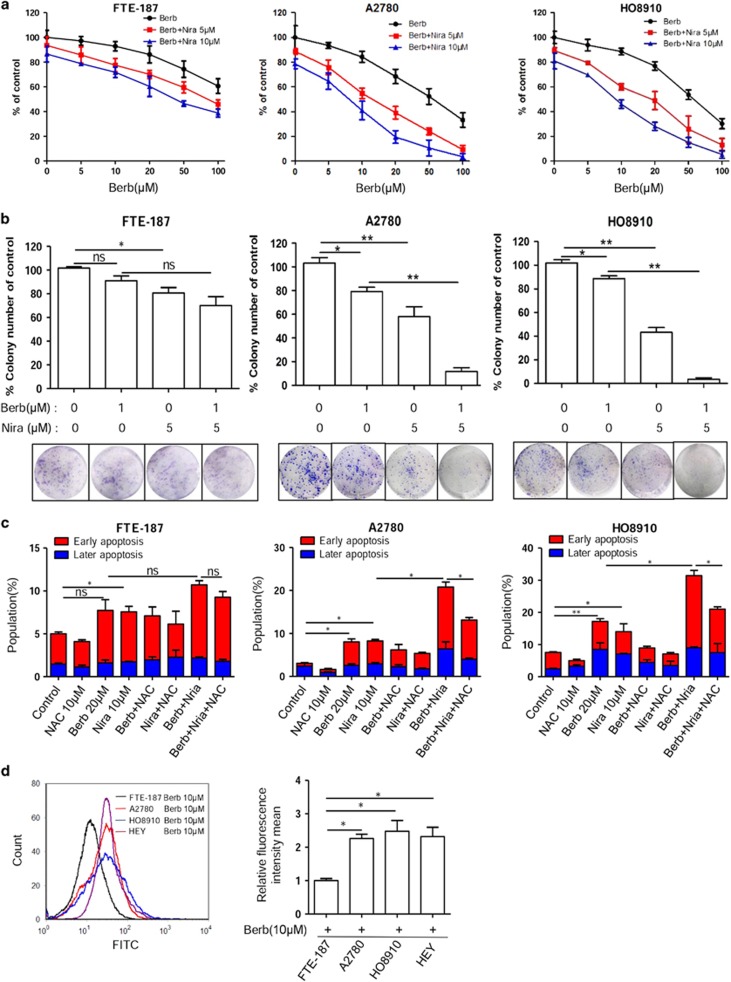
Berberine sensitizes ovarian cancer cells to PARP inhibitor by induction of apoptosis. (**a**) Cytotoxic effect of berberine alone or in combination with different concentrations of niraparib on ovarian cancer cells (FTE-187, A2780 and HO8910) measured by MTT assay. (**b**) Clonogenic assay showed inhibitory effect of berberine alone or in combination of niraparib on FTE-187, A2780 and HO8910 cells. (**c**) FTE-187, A2780 and HO8910 cells were treated with berberine, NAC or niraparib alone or in combination for 48 h. Apoptosis was measured by flow cytometry with annexin V and PI staining. (**d**) Intracellular accumulation of berberine in FTE-187, A2780, HO8910 and HEY cells, as measured by flow cytometry under 488 nm excitation. Cells were incubated in culture medium containing 10 *μ*M berberine for 48 h. *P<0.05, **P<0.01

Because berberine treatment increased oxidative stress and downregulated HR, conditions in which cancer cells would be more reliant on PARP for survival and proliferation, we next tested whether a combination of berberine and PARP inhibitor would be more effective in killing ovarian cancer cells by using MTT viability assay, colony formation assay and flow cytometry analysis of apoptotic cells. As shown in Figure 5a, whereas berberine induced a dose-dependent decrease in the viability of A2780 and HO8910 cells, addition of niraparib accelerated the decrease. Consistently, whereas colony formation was reduced by berberine and niraparib when each was applied alone, the reduction was more drastic when the two were applied in combination (Figure 5b, *P*<0.05 for A2780; *P*<0.01 for HO8910). Furthermore, the induction of apoptosis by the two drugs in combination was far greater than what would be caused by the additive effect of the two (Figure 5c, *P*<0.01 for both cell lines). Together, these results indicate that the two drugs act in synergy in exerting their proliferation-inhibiting effect.

Sensitization to PARP inhibition is generally believed to be dependent on impairment in homologous recombination. Because PARP1 is also involved in the repair of oxidative DNA damage, we next tested whether oxidative stress induced by berberine also contributes to the increased sensitivity of cancer cells to PARP inhibition. Indeed, when NAC was applied together with berberine and niraparib, apoptosis was significantly reduced (Figure 5c, *P*<0.05 for both A2780 and HO8910 cell lines), indicating that the synergistic effect of berberine and niraparib on cancer cells may also be mediated by oxidative DNA damage.

The induction of oxidative DNA damage by berberine was minimal in FTE-187 cells when compared with ovarian cancer cells (Figures 1a and 2a). Berberine did not further reduce the RAD51 level (Figure 3a). Consistently, a synergistic cytotoxic effect of berberine and niraparib was not detected in FTE-187 cells (Figures 5b and c). Because intracellular accumulation of berberine can be evaluated by its emission of green yellowish fluorescence,^6^ we next explored whether the less, or lack of, induction of oxidative DNA damage and RAD51 downregulation by berberine in FTE-187 cells was correlated to a reduced uptake/accumulation of berberine. Indeed, flow cytometry analysis showed a much reduced level of berberine accumulation in FTE-187 cells when compared with ovarian cancer cells (Figure 5d). These results indicate that the sensitizing effect of berberine is probably dependent on its cellular accumulation and the subsequent induction of oxidative stress and downregulation of HR.

### Therapeutic effect of berberine and niraparib in combination *in vivo*

**Figure 6:**
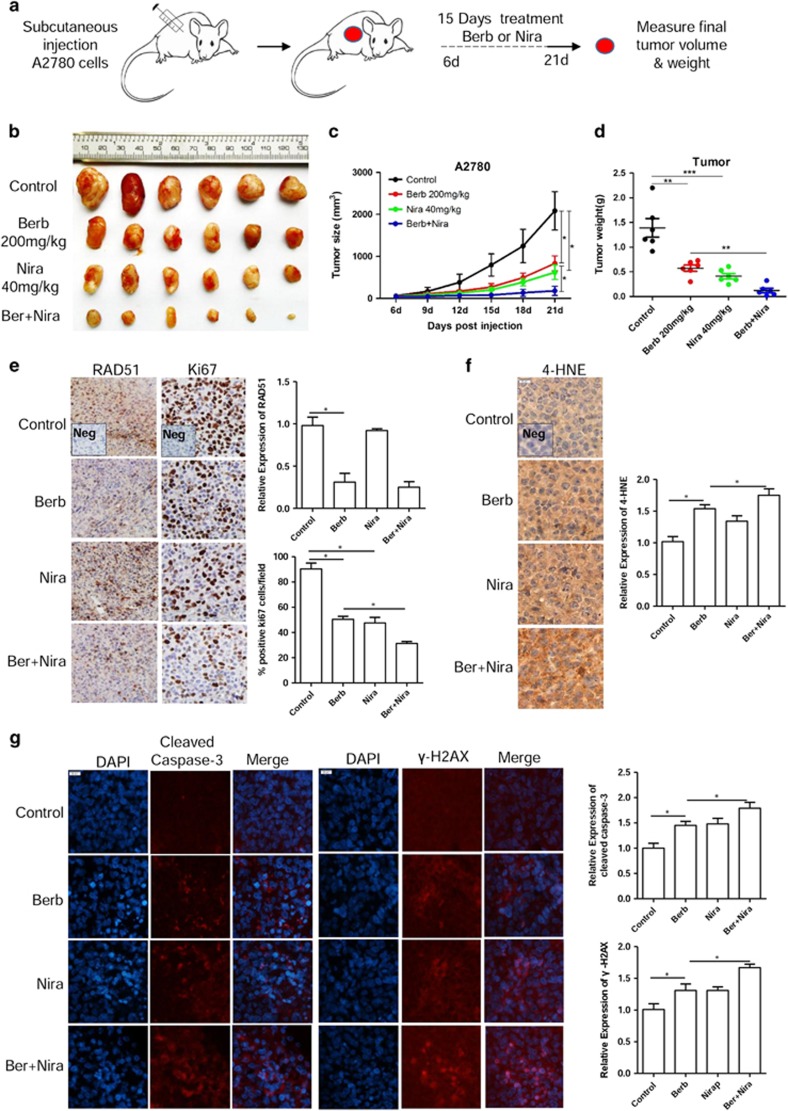
Combination of berberine and PARP inhibitor impedes tumor growth *in vivo*. (**a**) Scheme for the treatment paradigm. Mice were randomized into one of four groups; vehicle only (*n*=6), 200 mg/kg berberine only (*n*=6), 40 mg/kg niraparib only (*n*=6) or 200 mg/kg berberine plus 40 mg/kg niraparib (*n*=6). Tumor volumes were measured every 3 days and final weights were taken on day 21. (**b**) Images of A2780 tumors for each treatment group. (**c**) Growth curves of tumors from transplanted A2780 cells in nude mice for each treatment group. (**d**) Average tumor weight on day 21 for each treatment group. (**e**) Left: representative IHC images showing the RAD51 and Ki67. Scale bar, 20 *μ*m. Right: quantification of RAD51 expression and Ki67-positive cells in tumors for each treatment group. (**f**) Left: representative IHC images showing the 4-HNE. Scale bar, 20 *μ*m. Right: quantification of 4-HNE expression in tumors for each treatment group. (**g**) Left: representative IF images showing the cleaved caspase-3 and *γ*-H2AX; DAPI was used for the nuclear staining. Scale bar, 20 *μ*m. Right: quantification of cleaved caspase-3 and *γ*-H2AX expression in tumors for each treatment group. Immunohistochemistry intensities were quantified by ImageJ. *P<0.05, **P<0.01, ***P<0.001

We next tested the effect of berberine and niraparib on tumor growth *in vivo*. Tumor xenografts were generated by subcutaneously injecting A2780 ovarian cancer cells into nude mice. Six days later the tumor-bearing mice were randomly divided into four groups and were then treated daily by oral gavage with vehicle (sodium carboxymethylcellulose, SCMC), berberine, niraparib and berberine+niraparib, respectively, for 15 days (Figure 6a). As shown in Figures 6b–d, whereas berberine and niraparib each significantly inhibited tumor growth, combination of berberine and niraparib had more drastic effect. We also observed a remarkable reduction in RAD51 in tumor xenografts in berberine-treated hosts (Figure 6e). Consistent with the drastic reduction in the growth rate of xenografts, the percentage of Ki67-positive cells was greatly reduced in the berberine+niraparib group (Figure 6e). Moreover, the level of 4-hydroxynonenal (4-HNE), which reflects lipid peroxidation, was significantly induced by berberine (Figure 6f). Furthermore, significant induction of apoptosis, as measured by staining for cleaved caspase-3, was observed in all three treatment groups (Figure 6g). Significant increase in the level of DSBs, as evaluated by immunofluorescence staining for *γ*-H2AX, was also detected in tumor xenografts from treated mice (Figure 6g). These changes *in vivo* were consistent with those observed with the *in vitro* assays and further support the greatly increased potency when the two drugs were applied in combination.

## Discussion

Primary ovarian cancer is responsive to treatment, but chemoresistant recurrent disease ensues in the majority of patients.^3^ Novel strategies that improve chemosensitivity while minimizing undesirable side effects are needed to improve quality of life and therapeutic outcomes for ovarian cancer patients. In the present study, we investigated the therapeutic effect of berberine in combination with a PARP inhibitor on ovarian cancer cells and on tumor xenografts. We first confirmed that, as in other types of cancer cells, berberine was able to induce oxidative DNA damage and to downregulate RAD51 in ovarian cancer cells, two conditions that would render the cancer cells more reliant on PARP for survival and proliferation. As expected, berberine and niraparib indeed acted synergistically in killing ovarian cancer cells. Combination of the two drugs also drastically inhibited the growth of tumor xenografts formed by ovarian cancer cells. These results indicate that, in addition to having a direct antitumor effect, berberine also enhances the sensitivity of cancer cells to PARP inhibitors.

PARP inhibitors have been widely tested in clinical trials, and were shown to be particularly effective against cancers that are defective in HRR.^18, 19^ PARP primarily functions in the repair of single-strand breaks (SSBs). When PARP is inhibited, more SSBs would be converted into DSBs during the S phase. DSBs in the S phase are primarily repaired by HRR, and, if not repaired, as in the case of BRCA1/2-deficient cells, would lead to cell death. Therefore, PARP functional failure and HRR defect are synthetically lethal. PARP inhibition is a particularly attractive strategy for the management of ovarian cancer because HRR defects are common. However, for many types of cancer in which HRR is fully functional, the application of PARP inhibitors may be limited. Some recent studies showed that HRR could be impaired by certain natural small molecules, such as curcumin, berberine and artesunate.^14, 25, 26^ Those small molecules can potentially extend the application of PARP inhibitors to malignancies in which HRR is not intrinsically defective. Importantly, because berberine and artesunate also act as inducers of oxidative DNA damage, which is primarily repaired by base excision repair involving PARP1, their sensitizing effect may also be mediated by induction of oxidative stress. A search of COSMIC (the Catalog of Somatic Mutations in Cancer, http://cancer.sanger.ac.uk/cosmic) revealed no BRCA mutation in A2780, HEY, OVCAR3 and SKOV3 cell lines. HO8910 cell line carries BRCA1 5382 C mutation and is presumably HR-defective. Because berberine was able to sensitize both A2780 and HO8910 cells to niraparib, its pharmacological effect is probably independent of the intrinsic HR status of the cancer cell lines.

It should be noted that berberine is not known to have serious side effects in human. In fact, berberine is a common OTC herbal medicine for gastrointestinal discomfort in China. As shown in this study, the cytotoxic effect of berberine on FTE-187 cells is much smaller than that on ovarian cancer cells, which corresponds to reduced induction in ROS and oxidative DNA damage. Therefore, berberine as a sensitizer of cancer cells to PARP inhibitors should be further explored.

## Materials and methods

### Reagents and antibodies

Berberine chloride, 3-(4, 5-dimethylthiazol-2-yl)-2, 5-diphenyltetrazolium bromide (MTT) were purchased from Sigma (St. Louis, MO, USA). Niraparib (MK-4827) was purchased from Selleck (Houston, TX, USA). NAC was purchased from Beyotime Institute of Biotechnology (Haimen, China). Berberine chloride and niraparib were dissolved in DMSO and MTT was dissolved in phosphate-buffered solution (PBS). MitoSOX Red Mitochondrial Superoxide Indicator was purchased from Life Technologies (Carlsbad, CA, USA). Antibodies against RAD51 (H-92), *β*-actin (AC-15) and 8-OhdG (E-8) were purchased from Santa Cruz Biotechnology Inc. (Dallas, TX, USA) Anti-PAR was purchased from Calbiochem (Billerica, MA, USA). Anti-4-HNE (ab480506) was purchased from Abcam (Cambridge, UK). Anti-*γ*-H2AX (Ser139) was purchased from Upstate Biotechnology Inc (Lake Placid, NY, USA). Anti- ATM (D2E2), p-ATM (Ser1981), p53 (7F5), p-p53 (Ser15), Ki67 (9129S) and cleaved caspase-3 (Asp175) were from Cell Signaling Technology (Danvers, MA, USA).

### Cell lines and cell culture

A2780, HEY and SKOV3 cells were from Sigma-Aldrich (St. Louis, MO, USA). HO8910, HO8910PM and OVCAR3 cells were from Shanghai Cell Bank, Chinese Academy of Sciences (Shanghai). Immortalized fallopian tube surface epithelial cell line FTE-187 was as previously described.^27^ A2780, HO8910 and HEY cells were cultured in DMEM medium (GIBCO, Waltham, MA, USA; Invitrogen, Carlsbad, CA, USA). SKOV3, HO8910PM and OVCAR3 cells were cultured in RPMI 1640 medium (GIBCO, Invitrogen). FTE-187 cells were maintained in cell culture medium consisting of 1 : 1 Medium199 (Sigma-Aldrich) and MCDB105 medium (Sigma-Aldrich). All media contained 10% FBS (GIBCO, Invitrogen), 100 *μ*g/ml penicillin and 100 *μ*g/ml streptomycin. All cells were cultured in a humidified atmosphere of 5% CO_2_ at 37 °C.

### Quantification of intracellular ROS

Cells were washed and harvested in PBS and 10^6^cells were stained in 500 *μ*l PBS with 10 mM DHE bromide (Reagent B; Genmed Scientifics Inc., Wilmington, DE, USA) for 20 min at 37 °C in the dark. Samples were subsequently washed using ice-cold PBS and centrifuged for 10 min at 1000 r.p.m. before being resuspended in FACS dissociation solution (FACSmax, Genlantis, San Diego, CA, USA) and kept on ice until analysis. Flow cytometry was performed using the FACS-Calibur (Becton Dickinson Bioscience, Franklin Lakes, NJ, USA) counting a minimum of 10^4^ cells per sample. All experiments were repeated independently at least three times.

### Immunofluorescence staining of phospho-H2AX, 8-OHdG, RAD51 and PAR

Immunofluorescence staining was carried out as described previously.^6^ Briefly, cells grown on coverslips were fixed in 4% paraformaldehyde for 10 min. The cells were then permeabilized in 0.2% Triton X-100 for 10 min, and blocked in 10% normal goat serum overnight at 4 °C. The coverslips were incubated with anti-phospho-H2AX, 8-OhdG, RAD51 or PAR antibody overnight at 4 °C, washed in PBS and incubated with TRITC-conjugated Goat anti-mouse secondary antibody (Jackson ImmunoResearch Laboratories, West Grove, PA, USA) for 1 h at room temperature. Cells were washed in PBS three times and counterstained with DAPI. Fluorescence images were captured under a fluorescence microscope.

### Western blotting

Cells were lysed and protein concentrations were determined using the BCA assay. Protein samples were separated by SDS-PAGE (12%) and electrotransferred onto a PVDF membrane. The membrane was incubated with specific primary antibodies and appropriate horseradish peroxidase (HRP)-conjugated secondary antibodies. Then, proteins of interest were developed using ECL PLUS kit (Amersham Biosciences, Piscataway, NJ, USA). The protein levels were normalized by *β*-actin.

### Measurement of HRR

The GFP-based HRR reporter was as previously reported.^28^ Plasmids containing HR reporter cassettes were linearized by I-SceI restriction enzymes and purified using universal DNA purification kit (#DP214-03; Tiangen, Beijing, China). Two days after splitting, exponentially growing cells were transfected with 2 *μ*g of the HR reporter constructs and 0.1 *μ*g of pDsRed-N1 as the internal control, using lipofectamine 2000 Reagent. The lower amount of the pDsRed-N1 was used to avoid interference from the bright DsRed fluorescence. Fluorescence cells were scored using the FACS-Calibur (Becton Dickinson Bioscience).

### MTT assay of cell viability

MTT assay was carried out as described previously.^6^ Cells were seeded in 96-well plates at 3 × 10^3^/well the day before berberine treatment. Then, the cells were exposed to different doses of berberine for 24 or 48 h. After treatment, 20 *μ*l of MTT reagent, 5 mg/ml, was added to each well and incubated for 4 h at 37 °C. At the end of incubation, the media were carefully removed by aspiration. DMSO (100 *μ*l) was then added to each well. The plate was gently vortexed for 30 min at room temperature. The absorbance of each well was measured at 490 nm. All experiments were repeated at least three times.

### Clonogenic assay

**S**ingle-cell suspensions were generated for each cell line and specified numbers of cells were seeded into six-well tissue culture plates. Then, cells were exposed to different doses of berberine for 2 weeks. Colonies were stained with crystal violet. Colonies of greater than 50 cells were counted to determine the surviving fraction. The data presented are the mean±S.E. and represent three independent experiments.

### Apoptosis analysis

Ovarian cancer cells, treated with different doses of berberine, were harvested and washed once in cold PBS, and then stained with Alexa Fluor 488 annexin V and propidium iodide (PI; Alexa Fluor 488 annexin V/Dead Cell Apoptosis Kit with Alexa Fluor 488 annexin V and PI for Flow Cytometry, Invitrogen) and analyzed with flow cytometry using 488 nm excitation. Finally, the fraction of early apoptotic cells was determined with FCS Express V3 software (Glendale, CA, USA).

### Tumor xenografts

Six- to eight-week-old female nude mice were purchased from Beijing Experimental Animal Center and kept in pathogen-free conditions and handled in accordance with the requirements of the Guideline for Animal Experiments. The animals were subcutaneously inoculated with 1 × 10^6^ A2780 cells (suspended in 100 *μ*l PBS) and six days later the tumor-bearing mice were randomly divided into four groups and were then treated daily by oral gavage with vehicle (sodium carboxymethycellulose, SCMC), berberine (200 mg/kg bodyweight), niraparib (40 mg/kg bodyweight) and berberine (200 mg/kg) in combination with niraparib (40 mg/kg), respectively, for 15 days. Tumor growth was monitored with a caliper, and tumor volume was calculated according to the formula *V*=maximal diameter × perpendicular diameter.^2^

### Immunohistochemistry and immunofluorescence of tumor xenografts

After deparaffinization and rehydration, the sections were boiled in citrate sodium buffer for 15 min for antigen recovery, and were immersed in 3% H_2_O_2_ for 10 min to quench endogenous peroxidase. Sections were then blocked with 10% serum at 37 °C for 1 h. The primary antibodies (Ki67, CST, Danvers, MA, USA, 1 :  200 dilution; RAD51, Abcam, 1 : 200 dilution; 4-HNE, Abcam, 1 : 10 dilution; Cleaved Caspase-3, CST, 1 : 300 dilution; *γ*-H2AX, Upstate Biotechnology, 1 : 200 dilution) were added to the sections and incubated overnight at 4 °C. After washing, the sections (Ki67, RAD51 and 4-HNE) were coated with a HRP-conjugated second antibody (Jackson ImmunoResearch, West Grove, PA, USA; 1 : 200 dilution) and then incubated at 37 °C for 1 h. DAB was used to visualize immunoreactions. Sections (Cleaved Caspase-3, *γ*-H2AX) were then washed three times in PBS before incubating in the dark with a Rhodamine-labeled secondary antibody at a dilution of 1 : 300 in 5% bovine serum albumin in PBS for 60 min. The secondary antibody solution was then aspirated and the sections were washed four times in PBS. The sections were incubated in the dark with DAPI (1 mg/ml) in PBS for 5 min and coverslips were mounted with an antifade solution (Molecular Probes, Life Technologies, Carlsbad, CA, USA). Negative controls were performed by omitting the primary antibodies.

### Statistical analysis

All data are presented as mean and S.E.s in triplicate. Student's *t-*test was used for comparisons between two groups of experiments. The synergistic effect of berberine and niraparib was analyzed by ANOVA. *P*<0.05 was considered statistically significant.

## Publisher’s Note:

Springer Nature remains neutral with regard to jurisdictional claims in published maps and institutional affiliations.

## References

[bib1] Cho KR, Shih IeM. Ovarian cancer. Annu Rev Pathol 2009; 4: 287–313. 18842102 10.1146/annurev.pathol.4.110807.092246 PMC2679364

[bib2] Gamarra-Luques CD, Hapon MB, Goyeneche AA, Telleria CM. Resistance to cisplatin and paclitaxel does not affect the sensitivity of human ovarian cancer cells to antiprogestin-induced cytotoxicity. J Ovarian Res 2014; 7: 45. 24795781 10.1186/1757-2215-7-45 PMC4007005

[bib3] Vaughan S, Coward JI, Bast RC Jr., Berchuck A, Berek JS, Brenton JD et al. Rethinking ovarian cancer: recommendations for improving outcomes. Nat Rev 2011; 11: 719–725. 10.1038/nrc3144 PMC3380637 21941283

[bib4] Kong W, Wei J, Abidi P, Lin M, Inaba S, Li C et al. Berberine is a novel cholesterol-lowering drug working through a unique mechanism distinct from statins. Nat Med 2004; 10: 1344–1351. 15531889 10.1038/nm1135

[bib5] Tang J, Feng Y, Tsao S, Wang N, Curtain R, Wang Y. Berberine and Coptidis rhizoma as novel antineoplastic agents: a review of traditional use and biomedical investigations. J Ethnopharmacol 2009; 126: 5–17. 19686830 10.1016/j.jep.2009.08.009

[bib6] Liu Z, Liu Q, Xu B, Wu J, Guo C, Zhu F et al. Berberine induces p53-dependent cell cycle arrest and apoptosis of human osteosarcoma cells by inflicting DNA damage. Mutat Res 2009; 662: 75–83. 19159633 10.1016/j.mrfmmm.2008.12.009

[bib7] Goto H, Kariya R, Shimamoto M, Kudo E, Taura M, Katano H et al. Antitumor effect of berberine against primary effusion lymphoma via inhibition of NF-kappaB pathway. Cancer Sci 2012; 103: 775–781. 22320346 10.1111/j.1349-7006.2012.02212.x PMC7659260

[bib8] Wang Y, Liu Q, Liu Z, Li B, Sun Z, Zhou H et al. Berberine, a genotoxic alkaloid, induces ATM-Chk1 mediated G2 arrest in prostate cancer cells. Mutat Res 2012; 734: 20–29. 22561209 10.1016/j.mrfmmm.2012.04.005

[bib9] Liu Q, Xu X, Zhao M, Wei Z, Li X, Zhang X et al. Berberine induces senescence of human glioblastoma cells by downregulating the EGFR-MEK-ERK signaling pathway. Mol Cancer Ther 2015; 14: 355–363. 25504754 10.1158/1535-7163.MCT-14-0634

[bib10] Katiyar SK, Meeran SM, Katiyar N, Akhtar S. p53 Cooperates berberine-induced growth inhibition and apoptosis of non-small cell human lung cancer cells *in vitro* and tumor xenograft growth *in vivo*. Mol Carcinog 2009; 48: 24–37. 18459128 10.1002/mc.20453

[bib11] James MA, Fu H, Liu Y, Chen DR, You M. Dietary administration of berberine or Phellodendron amurense extract inhibits cell cycle progression and lung tumorigenesis. Mol Carcinog 2011; 50: 1–7. 21061266 10.1002/mc.20690 PMC6004604

[bib12] Ho YT, Yang JS, Lu CC, Chiang JH, Li TC, Lin JJ et al. Berberine inhibits human tongue squamous carcinoma cancer tumor growth in a murine xenograft model. Phytomedicine 2009; 16: 887–890. 19303753 10.1016/j.phymed.2009.02.015

[bib13] Wang L, Cao H, Lu N, Liu L, Wang B, Hu T et al. Berberine inhibits proliferation and down-regulates epidermal growth factor receptor through activation of Cbl in colon tumor cells. PLoS ONE 2013; 8: e56666. 23457600 10.1371/journal.pone.0056666 PMC3573001

[bib14] Liu Q, Jiang H, Liu Z, Wang Y, Zhao M, Hao C et al. Berberine radiosensitizes human esophageal cancer cells by downregulating homologous recombination repair protein RAD51. PLoS ONE 2011; 6: e23427. 21858113 10.1371/journal.pone.0023427 PMC3152570

[bib15] El-Khamisy SF, Masutani M, Suzuki H, Caldecott KW. A requirement for PARP-1 for the assembly or stability of XRCC1 nuclear foci at sites of oxidative DNA damage. Nucleic Acids Res 2003; 31: 5526–5533. 14500814 10.1093/nar/gkg761 PMC206461

[bib16] Luo X, Kraus WL, On PAR. with PARP: cellular stress signaling through poly(ADP-ribose) and PARP-1. Genes Dev 2012; 26: 417–432. 22391446 10.1101/gad.183509.111 PMC3305980

[bib17] Curtin NJ. DNA repair dysregulation from cancer driver to therapeutic target. Nat Rev Cancer 2012; 12: 801–817. 23175119 10.1038/nrc3399

[bib18] Bryant HE, Schultz N, Thomas HD, Parker KM, Flower D, Lopez E et al. Specific killing of BRCA2-deficient tumours with inhibitors of poly(ADP-ribose) polymerase. Nature 2005; 434: 913–917. 15829966 10.1038/nature03443

[bib19] Farmer H, McCabe N, Lord CJ, Tutt AN, Johnson DA, Richardson TB et al. Targeting the DNA repair defect in BRCA mutant cells as a therapeutic strategy. Nature 2005; 434: 917–921. 15829967 10.1038/nature03445

[bib20] McCabe N, Turner NC, Lord CJ, Kluzek K, Bialkowska A, Swift S et al. Deficiency in the repair of DNA damage by homologous recombination and sensitivity to poly(ADP-ribose) polymerase inhibition. Cancer Res 2006; 66: 8109–8115. 16912188 10.1158/0008-5472.CAN-06-0140

[bib21] Hsu WH, Hsieh YS, Kuo HC, Teng CY, Huang HI, Wang CJ et al. Berberine induces apoptosis in SW620 human colonic carcinoma cells through generation of reactive oxygen species and activation of JNK/p38 MAPK and FasL. Arch Toxicol 2007; 81: 719–728. 17673978 10.1007/s00204-006-0169-y

[bib22] Meeran SM, Katiyar S, Katiyar SK. Berberine-induced apoptosis in human prostate cancer cells is initiated by reactive oxygen species generation. Toxicol Appl Pharmacol 2008; 229: 33–43. 18275980 10.1016/j.taap.2007.12.027

[bib23] Hannay JA, Liu J, Zhu QS, Bolshakov SV, Li L, Pisters PW et al. Rad51 overexpression contributes to chemoresistance in human soft tissue sarcoma cells: a role for p53/activator protein 2 transcriptional regulation. Mol Cancer Ther 2007; 6: 1650–1660. 17513613 10.1158/1535-7163.MCT-06-0636

[bib24] Gottipati P, Vischioni B, Schultz N, Solomons J, Bryant HE, Djureinovic T et al. Poly(ADP-ribose) polymerase is hyperactivated in homologous recombination-defective cells. Cancer Res 2010; 70: 5389–5398. 20551068 10.1158/0008-5472.CAN-09-4716

[bib25] Ogiwara H, Ui A, Shiotani B, Zou L, Yasui A, Kohno T. Curcumin suppresses multiple DNA damage response pathways and has potency as a sensitizer to PARP inhibitor. Carcinogenesis 2013; 34: 2486–2497. 23825154 10.1093/carcin/bgt240

[bib26] Wang B, Hou D, Liu Q, Wu T, Guo H, Zhang X et al. Artesunate sensitizes ovarian cancer cells to cisplatin by downregulating RAD51. Cancer Biol Ther 2015; 16: 1548–1556. 26176175 10.1080/15384047.2015.1071738 PMC5391513

[bib27] Shan W, Mercado-Uribe I, Zhang J, Rosen D, Zhang S, Wei J et al. Mucinous adenocarcinoma developed from human fallopian tube epithelial cells through defined genetic modifications. Cell Cycle 2012; 11: 2107–2113. 22592533 10.4161/cc.20544 PMC3368862

[bib28] Mao Z, Seluanov A, Jiang Y, Gorbunova V. TRF2 is required for repair of nontelomeric DNA double-strand breaks by homologous recombination. Proc Natl Acad Sci USA 2007; 104: 13068–13073. 17670947 10.1073/pnas.0702410104 PMC1941808

